# LASSO Regression Modeling on Prediction of Medical Terms among Seafarers’ Health Documents Using Tidy Text Mining

**DOI:** 10.3390/bioengineering9030124

**Published:** 2022-03-17

**Authors:** Nalini Chintalapudi, Ulrico Angeloni, Gopi Battineni, Marzio di Canio, Claudia Marotta, Giovanni Rezza, Getu Gamo Sagaro, Andrea Silenzi, Francesco Amenta

**Affiliations:** 1 Clinical Research Centre, School of Medicinal and Health Products Sciences, University of Camerino, 62032 Camerino, Italy; gopi.battineni@unicam.it (G.B.); marzio.dicanio@unicam.it (M.d.C.); getugamo.sagaro@unicam.it (G.G.S.); francesco.amenta@unicam.it (F.A.); 2 General Directorate of Health Prevention, Ministry of Health, 00144 Rome, Italy; u.angeloni@sanita.it (U.A.); c.marotta@sanita.it (C.M.); g.rezza@sanita.it (G.R.); a.silenzi@sanita.it (A.S.); 3 Research Department, International Radio Medical Centre (C.I.R.M.), 00144 Rome, Italy

**Keywords:** seafarers, text mining, lasso regression, disease mapping, correlations

## Abstract

Generally, seafarers face a higher risk of illnesses and accidents than land workers. In most cases, there are no medical professionals on board seagoing vessels, which makes disease diagnosis even more difficult. When this occurs, onshore doctors may be able to provide medical advice through telemedicine by receiving better symptomatic and clinical details in the health abstracts of seafarers. The adoption of text mining techniques can assist in extracting diagnostic information from clinical texts. We applied lexicon sentimental analysis to explore the automatic labeling of positive and negative healthcare terms to seafarers’ text healthcare documents. This was due to the lack of experimental evaluations using computational techniques. In order to classify diseases and their associated symptoms, the LASSO regression algorithm is applied to analyze these text documents. A visualization of symptomatic data frequency for each disease can be achieved by analyzing TF-IDF values. The proposed approach allows for the classification of text documents with 93.8% accuracy by using a machine learning model called LASSO regression. It is possible to classify text documents effectively with tidy text mining libraries. In addition to delivering health assistance, this method can be used to classify diseases and establish health observatories. Knowledge developed in the present work will be applied to establish an Epidemiological Observatory of Seafarers’ Pathologies and Injuries. This Observatory will be a collaborative initiative of the Italian Ministry of Health, University of Camerino, and International Radio Medical Centre (C.I.R.M.), the Italian TMAS.

## 1. Introduction

Seafarers are regularly on the move and working at sea. As a result of long-term voyages, many international seafarers are away from their friends and families for at least six months per year [1]. The shipping industry is popular and used by 88% of world trade. However, it has a higher mortality rate, an injury rate, and a disease rate than land-based workers [2]. There are many risks associated with working at sea, including climate changes, seawater, humidity, and sun exposure [3,4]. Seafarers’ health and living conditions are affected by their working environment. Medical issues onboard are explained via telephone or internet by the captain in consultation with Telemedical Maritime Assistance Services (TMAS) doctors onshore. Health services are provided based on the severity of the case. Later, patient data are stored as digital health documents, which are created as text records [5,6].

Data analysts including those from different fields are often tasked with analyzing text or other unstructured data [7]. From the seafarers’ telemedicine data, we can extract pathology information, symptoms information, patient condition info, prescriptions from doctors, etc. We can use these data to make better medical decisions. For doing, we need advanced computational tools and skills to analyze unstructured text [8]. One way to learn more is to use word frequency analysis. Sentiment analysis allows one to extract mapping words and emotions from symptomatic words. As a result, we can develop a basic understanding of Text Mining (TM) approaches, which are widely applied to retrieve symptomatic data [9]. Medical pattern recognition software that converts texts to natural language using medical directories, algorithms, and information knowledge [10,11]. The use of TM techniques has proven to be beneficial in detecting depression symptoms with comprehensive diagnostic accuracy, according to studies in mental health [12]. According to [13], the integration of TM knowledge with neurological data can be used to detect neurological diseases and syndromes based on annual data.

Natural language processing (NLP) and machine learning (ML) techniques are used in sentiment analysis (also known as opinion mining) [14]. Medical sentimental analysis is used for the evaluation of medical records and automated decision support systems [15,16], as well as for assessing a patient’s emotions and sentiments based on medical history [17]. Likewise, a hybrid text model has been designed to help healthcare workers diagnose diseases such as diabetes and dementia [18,19]. Social media and the web can be analyzed with TM to identify knowledge about diabetes diagnosis and treatment. In this study, patterns in the web and social media text were analyzed to discover previously overlooked diagnoses or conditions of diabetes via comparison with those from standard diabetes treatment and diagnosis [19].

The literature on TM primarily focuses on sentiment analyses, such as emotion recognition, handwritten/typed text analysis, and data visualization. The authors of [20] provided a list of all methodologies and approaches for performing sentiment analysis based on three categories: machine learning, dictionaries, and ontologies. The study provides a brief introduction to opinion mining issues such as data sparsity, binary classification, and polarity shift problems in distinct domains. Various analytical algorithms are discussed to extract the text from mixed documents with the typed and handwritten text [21]. The study examines the sentimental analysis of medical treatment in detail and shows new challenges and possibilities in the medical field [22].

A previously published study [9] was extended in the present paper to highlight the importance of tidy TM in presenting symptomatic words of diseases common to seafarers. To represent the patient’s emotions or feedback, we applied sentimental analysis to medical abstract documents with disease names and symptoms. Seafarers’ medical documents are not aligned with the TM documentation. By mapping tidy TM symptom words to common diseases occurring onboard, we attempted to circumvent this limitation. The present study is the first comprehensive analysis of seafarers’ diseases that has been conducted by researchers who have an understanding of maritime medicine.

We can understand a subject’s emotions and behavior when studying such medical documents with text since emotions play an active role in human behavior. A domain-specific corpus from the digital health database contains clinical documents, which is critical for decision-making [15]. Text mining algorithms are applied to text containing seafarers’ medical documents in the analysis of text containing TM of telemedical documents. The least absolute shrinkage and selection operator (LASSO) regression models were applied for text classification as well as for defining word relevance through this variable selection.

## 2. Materials and Methods

Methods used for the analysis included document collection, pre-processing, and sentiment analysis. The frequency of disease terms was determined by analyzing sentiment in the Bing lexicon. By using these techniques, we estimated the most commonly used words among seafarers.

### 2.1. Data Collection

Medical text data of seafarers were examined from 2006 to 2021 and data was analyzed among 41,292 seafarers who got telemedical assistance through the International Radio Medical Centre (C.I.R.M.). The Centre establishes digital medical files for each case after it makes contact with the ship and updates them and this study analysed these files.

Data for the last 15 years (2006–2021) were extracted from 41,292 text documents containing patient information, a seafarer sending a message (Tx), and a doctor receiving a message (Rx). Messages TX include ship name and radio call sign, position, destination port, estimated time of arrival, course, speed, patient’s age, nationality, qualification, vital signs like breathing, pulse, temperature, and blood pressure, symptoms of localized pain, medical history, and medicines available on board. The message Rx contains a doctor’s questions, treatment, diagnosis, and diet and prevention instructions, as well as all the patient’s treatment information.

Documents include text data such as symptomatic information, doctor prescriptions, treatment information, medication details, etc. C.I.R.M. physicians classified diagnoses according to the International Classification of Diseases (ICD)-10 (WHO, 2007). Health management, epidemiology, and clinical analysis rely on this standard. Table 1 provides an example of medical abstracts for treatments. For a smooth experimental setup in the R framework, all text data were prepared as a CSV document.

### 2.2. Corpus Pre-Processing

Data cleaning ensures that user data is consistent, reliable, and accurate, and the text should be organized logically, especially for in-text data. We come across questions regarding punctuation, abbreviations, and contractions after reading the corpus. The removal of stop words and stem words, as well as the treatment of lower- and uppercase letters, is also needed. In tidy TM, clean_corpus is a function within the tidytext package that helps process the corpus [23]. With some default tools, like strings (for text cleaning), this package can convert upper case letters into lower case.

### 2.3. Tidy Text Mining and Packages

We can manage text simply and easily by using tidy data standards. The tidy data structure has the variables as columns, observations as rows, and each observational unit type as a table [24]. Thus, a tidy text format appears as a table with one token per row. For text analysis, tokens are semantically meaningful words, and tokenization is the process of dividing text data into tokens.

**Figure 1:**
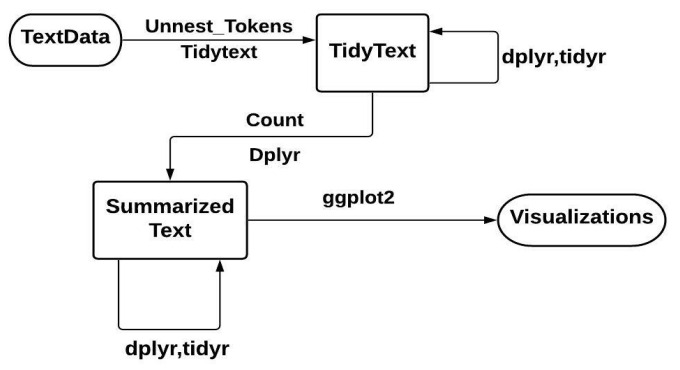
Flowchart representation of typical text analysis using principles of tidy data.

With tidy TM, the token is stored in every row, which can be a single word, a sentence, an n-gram, or a paragraph. The ‘tidytext’ package provides the functionality of tokenization with commonly encountered text units. In this package, there is no requirement that the user maintains a clean text format at all times. Using dplyr and other tidy tools, the text is processed, filtered, and imported, and then data is converted into a document-term matrix (DTM) for use in ML applications, and ggplot2 can then be used to visualize and interpret these models [25]. Figure 1 shows the flowchart representation with help of tidy data principles.

### 2.4. Sentimental Analysis

**Figure 2:**
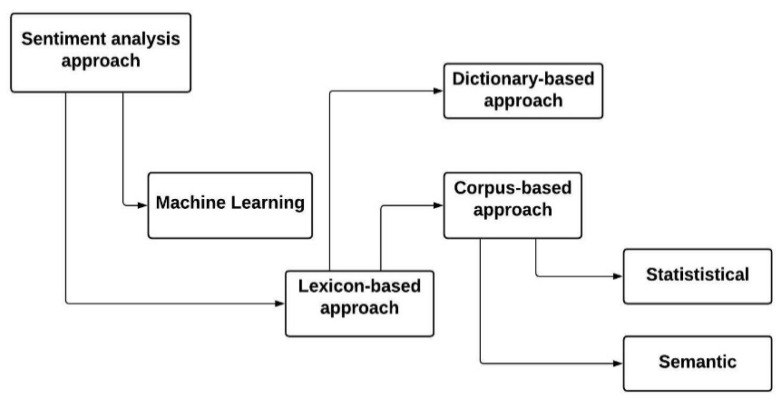
Lexicon-based sentimental analysis architecture for text documents.

In order to make product sales more effective, a company manager might want to find out if the product reviews are positive or negative. It is possible to examine text sentiment using a word’s combination and the sentimental content of the entire text by analyzing the sentiment of the singular words. With tidy TM, lexicon-based sentiment analysis is frequently used to calculate sentiment distributions based on lexicon alignments [26]. This semantic alignment can be negative, positive, or neutral. The lexicons dictionary can be formed either manually or automatically. Figure 2 illustrates the architecture of lexicon-based analysis.

A lexicon-based analysis determines the semantic orientation of the text by looking at adverbs and adjectives. This can be converted into a single score for the entire value in the final assessment. Three general lexicons can be found in tidy TM, namely AFINN, BING, and NRC. In the AFINN lexicon, sentimental scores range from −5 to 5, with negative scores for negative sentiment and positive scores for positive sentiment. As with the Bing lexicon, the NRC lexicon categorizes sentiments equally into yes/no categories as positive/negative.

### 2.5. Calculation of Word and Document Frequency (TF-IDF)

The term frequency-inverse document frequency (TF-IDF) indicates how relevant a medical term is to a particular telemedical document. A TF-IDF is a method for measuring the importance of a specific word in a document in comparison to the total number of documents. It is calculated as follows:

(1) $$\mathrm{TF}(t)=\frac{\mathrm{Number}\;\mathrm{of}\;\mathrm{times}\;\mathrm{term}\;t\;\mathrm{appears}\;\mathrm{in}\;a\;\mathrm{document}}{\mathrm{Total}\;\mathrm{number}\;\mathrm{of}\;\mathrm{terms}\;\mathrm{in}\;}$$

where TF(t) presents how often a particular term appears in a document. Similarly, the inverse document frequency (IDF) is a metric value representing the information provided by a particular word. Mathematically it is presented as a fraction of logarithmically inverse documents that contain the words. It is mathematically denoted as

(2) $$\mathrm{IDF}\;(t)=\log\;\frac{\mathrm{number}\;\mathrm{of}\;\mathrm{documents}}{\mathrm{number}\;\mathrm{of}\;\mathrm{documents}\;\mathrm{containing}\;\mathrm{term}}$$

Simply, Equation (2) is logarithm of document number in corpus (nominator) divided by number of documents where particular term appears (denominator). It is likely to get words such as ‘is’, ‘are’, ‘the’, and ‘an’ in the calculation of most frequent words in the corpus. By removing buzz and stop words from the medical abstracts, we will get words like ‘seafarers’, ‘accidents’, and ‘pathologies’. Thus, the TF-IDF metric measures the frequency of terms and weights them by how rarely they are used. The term frequency in medical documents refers to how frequently a particular term appears within an individual document. Seafarers’ medical records are often referred to as ‘frequency’ in general. The terms ‘seafarers’, ‘accidents’, and ‘pathologies’ occurred very often in this work, and they are very frequently used in a given document, resulting in its low rating for TF-IDF.

### 2.6. Word Clouds

One way to visualize the high probability words in the text using TM packages is by using word clouds [26]. The word clouds can also be called text clouds and are created with the TM package (tm), and word cloud creator package (word cloud) [27], both of which are available in R for helping visualize words in text quickly. In the word cloud, the size represents the frequency of words. They may appear like a visualization of popular positive and negative words, but the size of words cannot be compared to the sentiment they convey.

### 2.7. LASSO Regression Model

LASSO takes advantage of shrinkage to accomplish linear regression. When a data value shrinks towards a central point, such as a mean, shrinkage occurs. As a result, it is well suited to models with high levels of multicollinearity. It also allows automated parts of model selection, such as parameter elimination and variable selection [28].

The LASSO regression model is being used more and more in medical diagnosis to predict disease outcomes and side effects. The model has been applied to brain modeling [29], biomarker selection [30], healthcare cost prediction [31], and early detection of cardiovascular diseases [32]. The classification of medical documents is widely used in healthcare, but few studies have been conducted on it. Our work demonstrates how LASSO is applied to text data using the principles of tidy data. This model extends supervised machine learning to text classification.

LASSO problems are quadratic programming problems that aim to minimize. In statistics, it was written as

(3) $$\overset{n}{\underset{i\;=1}{\sum }}\left(\mathrm{yi}-\underset{j}{\sum }x_{\mathrm{ij}}\beta_{j}\right)2+ƛ\overset{p}{\underset{j\;=1}{\sum }}\left|\beta_{j}\right|$$

The above equation is the same as the minimization of the sum of squares with constraint $\Sigma \left|\beta_{j\;}\right|$ ≤ s. To interpret the model easily, some of β s can be shrunk to almost zero and results regression model to do easy interpretation. Here ƛ is a tuning parameter (i.e., amount of shrinkage). When ƛ = 0, no parameters are eliminated. When it increases, bias increases and when it decreases, variance increases.

### 2.8. Model Training and Evaluation

After the data were ready, they were divided into training and testing sets. The data are used both for building the model and evaluating its performance. LASSO regularization was applied for our logistic regression model with the glmnet package, and it can help to detect keywords in a prediction. We compare the LASSO model performance with two supervised models, namely Support Vector Machines (SVM) [33] and Random Forest (RF) [34]. We then validate the model using cross-validation (CV). As a resampling method for statistical analysis, this approach is known as rotation estimation. Therefore, in order to implement the CV technique, the data sample is segmented into different subsets. The analysis is performed on a subset called a training set. The results are then verified on the other subset called a testing set or a validation set [35]. By creating a data frame that can be displayed to each document in the dataset, the model’s performance is evaluated by applying tidy data principles. Based on the percentage of correctly classified outcomes over the total outcomes, classification performance is calculated.

## 3. Results

A benefit of using tidy data is sentiment analysis, which can be performed as an inner join. The ability to perform sentiment analysis as an inner join is another practical example of using TM as tidy analysis, similar to removing stop words as an antijoin operation.

### 3.1. Sentimental Analysis

**Figure 3:**
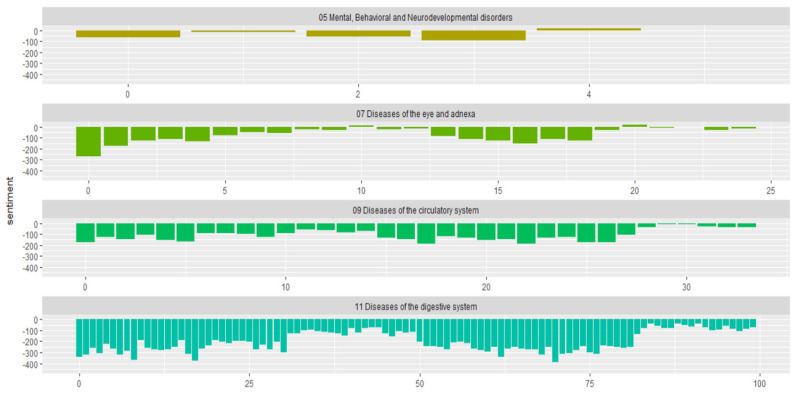
Lexicon based sentimental scores of the ICD 10 disease types (this is the plot of each disease sentiment changes towards more negative or positive over the times appearing in a dataset).

As part of our study, we examined how sentiments of each symptom varied across categorical diseases of ICD 10. We first determine the sentiment scores for each symptomatic word by using Bing lexicon and inner join functions. Figure 3 shows how sentimental scores (*Y*-axis) and the plotting of medical documents for certain diseases change and become more positive or negative over time (*X*-axis).

**Figure 4:**
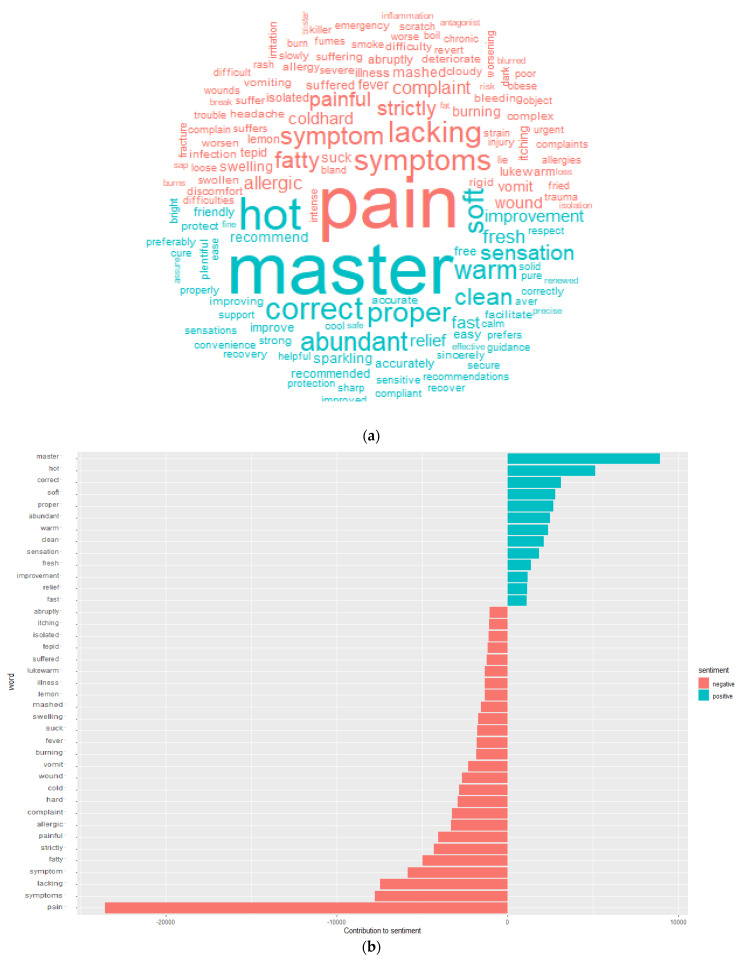
(**a**). Word cloud picturization of positive (green) and negative (red) sentimental words (most of the word alignments are associated with words pain, master, symptoms, hot, correct, lacking etc.). (**b**). Word count that contributes both negative and positive sentiments; the ‘pain’ word had the highest negative sentiment count (23,557) and the ‘master’ word has the highest positive sentiment count (8935).

The data frame includes both a word and a sentiment, so we can easily determine the number of words that contribute to each statement. Figure 4a shows sentiment visualization in the form of word clouds, while Figure 4b shows the word distribution count for positive and negative sentiments.

### 3.2. TF-IDF Calculation

**Figure 5:**
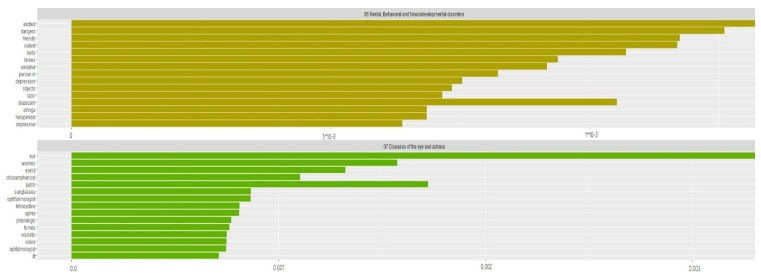
TF-IDF word count for mental health and eye diseases category; the highest frequency symptomatic words calculated by TF-IDF are vital to disease diagnosis. This outcome presents the proper distinguishment of keywords that are important to specific categorical documents within the collection in a group of documents.

By reducing the weight of commonly occurring words and boosting the weight of words that are less frequently used in the document corpus, TF-IDF identifies medical keywords associated with each disease category. We examined a large number of medical documents of seafarers, identifying symptoms of several disease categories. Using the ICD-10, we classified the documents into 22 groups. Most of the important words did not appear in all the categories. Figure 5 shows how disease documents categorize the major keywords. ICD code 05 (mental, behavioral and neurodevelopmental disorders) contains the keywords friendly, excited, dangerous, violent and depression with TF-IDF scores 0.001174, 0.001323, 0.001260, 0.001169 and 0.000754. Seafarers tend to suffer from anxiety and depression more often than onshore workers due to their long days away from their families [36]. As a result, tidy TM packages are able to identify a given disease’s most common symptoms.

### 3.3. Bigrams and Correlations

**Figure 6:**
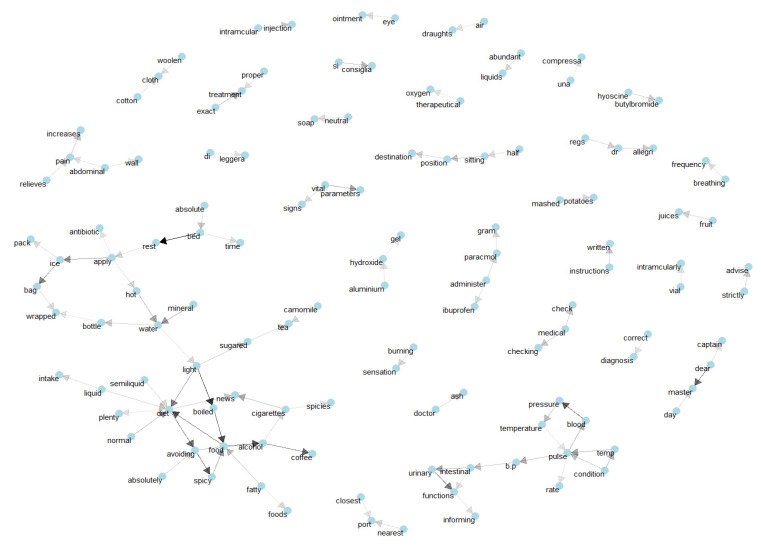
Data visualization networks (Common bigrams that occurred in categorical disease documents).

Using Bigrams, we can also show how medical words relate to one another. Bigrams are visual representations of words arranged in a graph or network. In this study, we considered a graph with multiple nodes. Graphs were created using the igraph package, which has powerful manipulation and analysis functions. In Figure 6, we can see a relationship between different words in the medical records of seafarers. The diet nodes are connected to words including fatty, spicy, semiliquid, coffee, cigarettes, spices, etc. There are also triplets with similar meanings (‘cloth’, ‘cotton’, or ‘woolen’).

**Figure 7:**
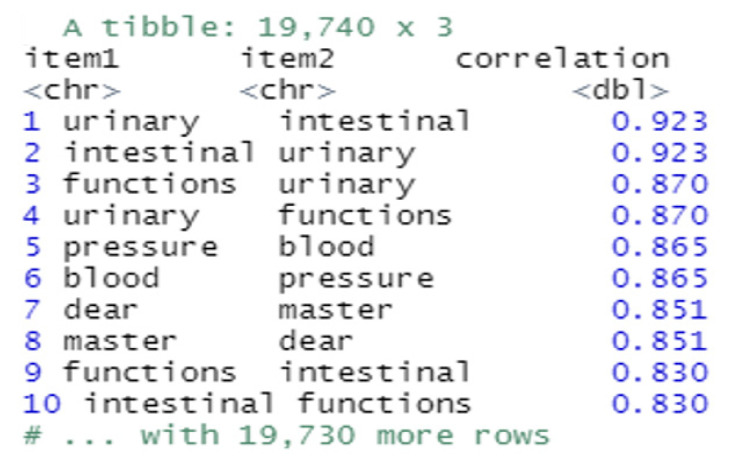
Correlation table between the symptomatic words.

Additionally, correlation establishes a link between dependent and independent words. Having a negative correlation indicates a decrease in what we are measuring. This enabled us to determine which words are closely related to a particular medical term. In this experiment, we choose some popular words and find other words that are most related to them (Figure 7). There is 92.3% correlation between the word ‘intestinal’ and the word ‘urinary’.

### 3.4. Text Classification with ML Modelling

From the large sample of medical documents, we selected two categorical documents, such as cardiovascular and digestive diseases. The document sample is 8803 and it is further divided into 70:30, where 70% of documents are for training and 30% are for test purposes. Three models were trained with an optimal parameter which was defined by CV validation. Each experiment was conducted with a 10-k value. The Receiver Operating Characteristics (ROC) curves can be used in medical diagnosis to test the model’s ability to predict text in documents [37]. In particular, ROC curves are known for their ability to visualize binary classification. In Table 2, we compare the performance metrics in terms of accuracy, sensitivity, specificity, and ROC.

**Figure 8:**
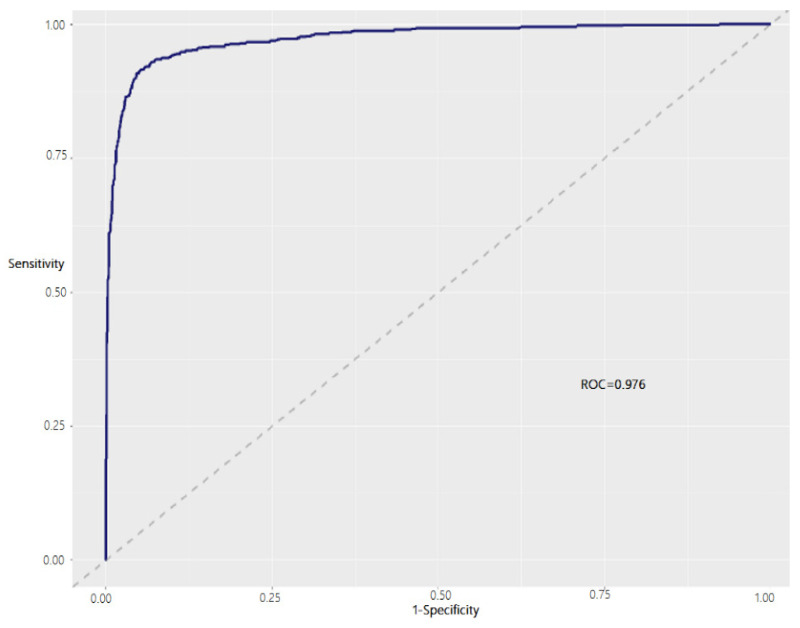
ROC curve for text classification using LASSO regularized regression.

The accuracy can be measured as the ratio between some of the true predicted documents and the total number of documents. Among the 2641 documents tested, the LASSO model correctly predicted 2477 documents, while 164 were incorrectly predicted, resulting in a 93.8% accuracy. The Figure 8 presents the ROC curve outcome for LASSO regression model where false positive rate (1-specificity) on *x*-axis and true positive rate (sensitivity) on *y*-axis. It is obvious that the ROC value of 0.976 shows a perfect classification of the categorical documents included. These results indicate that LASSO regression outperforms the other two classification models. Moreover, sensitivity is the percentage of correctly predicted outcomes, and this model scored 97.9%, a relatively high value for prediction problems.

## 4. Discussion

This paper presents an integrated analysis of medical documents of seafarers that incorporates TM and sentiment analysis. Due to sailors’ distance from medical facilities, health care is difficult. When doctors onshore diagnose correctly, they can prescribe effective treatments [38,39,40]. Using electronic health records (EHRs) can help reorganize services, train staff on the benefits of working with different teams, and protect staff health, according to this study. A lack of assistance will put staff and seafarers at risk.

Seafarers often suffer from swelling, fatigue, general weakness, headaches, confusion, and other symptoms. Different studies have found a connection between symptoms, including hemoptysis, thrombocytopenia, and high C-reactive protein levels [41,42]. A hypoxemic patient can develop acute respiratory distress syndrome (ARDS), sepsis, and even multi-organ failure in a short amount of time [43]. A seafarer’s disease type must be diagnosed quickly and treated according to his symptomatology.

Medical care professionals can utilize TM to collect and write information from the patient’s EHRs to support decision-making and disease diagnosis [44]. Pattern-based mining from EHRs assists doctors, physicians, and laboratory experts by retrieving significant knowledge from the system [45]. Pletscher-Frankild and coworkers developed a TM software program that identifies human and disease genes in text and identifies disease-gene associations [46]. A study analyzed 100 posts from epilepsy patient forums to quantitatively analyze patient perspectives on treatments, which may aid medical experts in designing clinical decision making based on patient-derived information [47].

In recent reports, the importance of EHRs to the functioning of healthcare systems has been emphasized [48]. Seafarers, for example, have been provided with telematics services for years. Medical prescriptions are issued in a single, standardized format. Physicians and pharmacies are required to submit prescription data electronically. Online medical records are preferred by doctors when prescribing treatments. Text information is stored in these records. These types of data can be processed by healthcare experts, but they can only estimate which diseases they are dealing with.

TM has been used to predict hospital admissions based on emergency department initial medical records [49]. TM can provide valuable information and make it easier for bed management teams to make decisions. It is reported that TM also plays an influential role in the autonomous classification of hospital admissions. Moreover, it is reported that TM examines the performance of text classification from clinical data from hospital reports [50]. This describes how TM can identify hospitalized patients for the treatment of a given disease based on the information associated with patient admission. This study used a machine learning approach called LASSO regression to distinguish disease documents based on clinical terms. The application of statistical and epidemiological methods in medical research was done with TM. The authors in [51], review the last twenty-year reports to identify commonly encountered and emergent methods used to investigate medical research problems.

In medical research, TM was applied to statistical and epidemiological analyses. Meaney and colleagues reviewed reports from the last twenty years to identify commonly encountered and emerging research methods [51]. The TM has been used to predict hospital admissions using emergency department records [49]. Providing this information will help bed management teams make better decisions. TM is also utilized to classify hospital admissions as well as hospital reports [50]. The information is derived from the admission information for the patient. Through TM, patients can be identified for specific diseases.

In an emergency situation, TM knowledge is extremely valuable because it allows high-quality data to be generated in real time. It is imperative for patients and medical staff to be careful when providing information [52]. Therefore, a gap can develop between the data scientists, scholars, and medical professionals capable of producing the data. The importance of sharing data across care providers will also be affected by this gap. A tidy TM needs to be presented in these situations so that raw data can be visualized in a prescribed format and distributed evenly among specialists. Medical abstracts have been organized neatly in this paper, and symptom maps for illnesses experienced onboard have been visualized. LASSO regression models are also used to validate the results. Remote doctors provide maritime telemedicine assistance, but these practices illustrate the limitations of using digital health records to produce quality data.

## 5. Conclusions

For the management of unstructured datasets, tidy-based TM has proven to be a comprehensive and efficient tool. It is relatively difficult to recognize treatments and relevant facts in medical documents written in languages other than English. By combining tidy TM packages and libraries with semantic manipulation, we developed a comprehensive approach to identifying onboard diseases. An ICD-10 symptom mapping was also undertaken. Symptom correlation plots, which measure how different health problems are linked together, were also presented. Using LASSO regression, this study successfully predicted text data among documents with 93.8% accuracy. These tidy TM libraries can effectively classify text documents in healthcare analysis projects. As well as delivering medical assistance, this approach may be used to develop health observatories and to classify diseases. We propose to apply the knowledge developed in this work to the Epidemiological Observatory of Seafarers Pathologies and Injuries, a collaborative initiative between the Ministry of Health, University of Camerino, and the International Radio Medical Center (C.I.R.M.).

## Figures and Tables

**Table 1:** Sample of medical abstracts and treatment of given diagnosis.

Year	Case Number	Diagnosis	Medical Abstract	Suggested Treatment
2006	88	Abdominalgia	Mild pain in the lower part of the stomach and temperature.	Discontinue aspirin. Keep patient bed rest in the most comfortable position. Apply an ice bag wrapped in a cotton cloth on the painful area if it relieves pain.
2008	17	Acute Gastritis	The patient said he has stomach pain; he has a history of hyperacidity.	Keep patient rest in a sitting position. Give buscopan one tablet every six hrs. give antacid every six hrs. Give omeprazole one tablet every twelve hrs. Light boiled food diet with a large intake of mineral water. Give news in twelve hrs.
2009	151	Allergic Reaction	The rash on a body appears in various places namely round an eye, bridge of the nouse, behind an ear, on a breast and a back, on a neck and hands.	Keep resting cotton loose-fitting clothes. continue ciprofloxacin milligram. Cetrizine or chlophenaramine. Boiled food diet with abundant water. Avoid all contact with cargo.
2013	597	Fever	Stomach pain with loose motions, mainly at night. Burning sensation during urination especially during evenings when the fever sets in.	Keep bed rest far from air draughts and extremes of temp. Apply ice bag wrapped in a cotton cloth on the head when temperature rises above 39 °C Continue Paracetamol, Ciprofloxacin, continue also Buscopan.
2014	1042	Haemorrhage	Patient with profuse blooding from yesterday at the gingival level (maybe the presence of abscess) and of the urinary tract. He has lost knowledge several times yesterday and today, already underway in fluid therapy.	Continue fluid therapy with Ranitidine fl inside the flexo, Tranexamic acid is not available onboard. Give as antibiotic Amoxicillin 1 g CPR if not allergic. Urgent disembarkation should be organized with a faster vehicle.
2016	197	Anxious-Depressive Syndrome	Please note that for the last two days the patient had been complaining of improper sleep. He reported that he was feeling a little depressed. He also reported that he does not feel capable of keeping navigational watches during hours of darkness as it gives him a feeling of loneliness.	Keep at rest in the bed or armchair as he prefers but, in any case, under continuous control by a friendly person. Remove from his cabin dangerous objects (knives, forks, glasses, razor blades, belts, shoelaces, dangerous drugs, gas lighters, anything through which he can injure himself or other people).
2020	746	Foreign Body	One of the people in the crew has swollen right eye. He got some foreign dust particles inside his eye, he rubbed his eye with his dirty hands, the eye started swelling and itching. We gave him an eyewash and suggested washing the eye regularly. Looks like due to rubbing the eye, he developed an eye infection. Kindly advise treatment we can give.	Keep rest not necessary in bed in a semi-dark room. Wash accurately’s the eye with sterile saline solution or e Optrex or other eye leashes. Then when dry apply eye antibiotic ointment and cover with a sterile or light bandage.
2021	54	Odontalgia	Complain regarding the patient’s tooth on the lower left molar. It was found out that the filling was been detached which causes pain.	Keep at rest. Apply inside the tooth cavity a small ball of cotton wool soaked in clove oil. Administer Paracetamol one 500 mg tablet every 6 h and Co-amoxiclav one gram tablet every 12 h. A light diet with easily chewable foods and a large intake of liquids.

**Table 2:** Performance comparison of adopted models (k = 10).

Model	Accuracy (%)	Sensitivity (%)	Specificity (%)	ROC
SVM	64.2	68.3	45.3	0.597
RF	59.0	59.8	55.4	0.613
LASSO	93.8	97.9	80.6	0.976

## Data Availability

Data examined for the present study were collected and stored in a closed database by the Centro Internazionale Radio Medico (C.I.R.M.), the Italian Maritime Telemedical Assistance Service (TMAS) in the frame of health surveillance activities performed onboard ships. Data were extracted from the database by C.I.R.M. operators and anonymized before being used for research purposes. C.I.R.M. President as legal representative of the entity where medical data are kept has authorized access to authors for collecting data of this work. The programing code for experiments that are involved can be found in https://github.com/nalinichintalapudi/Tidymodels-for-medical-text-data.git.
